# A shared somatic translocation involving *CUX1* in monozygotic twins as an early driver of AMKL in Down syndrome

**DOI:** 10.1038/s41408-020-0293-6

**Published:** 2020-03-03

**Authors:** Iben Bache, Karin Wadt, Mana M. Mehrjouy, Maria Rossing, Olga Østrup, Anna Byrjalsen, Niels Tommerup, Marlen Metzner, Paresh Vyas, Kjeld Schmiegelow, Birgitte Lausen, Mette K. Andersen

**Affiliations:** 1 Department of Cellular and Molecular Medicine, University of Copenhagen, Copenhagen, Denmark; 2 Department of Clinical Genetics, Copenhagen University Hospital, Rigshospitalet, Copenhagen, Denmark; 3 Department of Pediatrics and Adolescent Medicine, Copenhagen University Hospital, Rigshospitalet, Copenhagen, Denmark; 4 Centre for Genomic Medicine, Copenhagen University Hospital, Rigshospitalet, Copenhagen, Denmark; 5 MRC MHU, BRC Hematology Theme, Oxford Biomedical Research Centre, Oxford Centre for Haematology, WIMM, Radcliffe Department of Medicine, University of Oxford, Oxford, UK; 6 Department of Haematology, Oxford University Hospitals NHS Trust, Oxford, UK; 7 Institute for Clinical Medicine, Faculty of Medicine, University of Copenhagen, Copenhagen, Denmark

**Keywords:** Cytogenetics, Genetics research

Dear Editor,

Children with Down syndrome (DS) have an increased risk of developing leukemia^1^, especially acute megakaryoblastic leukemia (AMKL) preceded by a somatic *GATA1* mutation and often by the neonatal syndrome transient abnormal myelopoiesis (TAM)^2^. However, progression into AMKL occurs only in ~20% of the TAM patients^3^. Hence, the development of DS–AMKL can be described by three phases: (i) a disturbance of the natural fetal hematopoiesis due to the constitutional trisomy 21; (ii) a somatic *GATA1* mutation and in most patients TAM; (iii) progression into AMKL if additional somatic mutations occur in a dormant *GATA1*-mutated clone. The acquired mutations found in the third phase vary between patients, and can include cytogenetic abnormalities like loss of chromosome 7, gain of chromosomes 8, 14, and 21, duplication 1q^4,5^, and single-gene mutations, e.g., in the cohesin protein family, signaling molecules, and epigenetic regulators^6,7^. However, little is known about the developmental timing of the third-hit mutations, which are essential for the DS–AMKL progression.

Monozygotic twins concordant for a hematological disease with identical clonal mutations are rare but have been crucial in ascertaining the prenatal development of somatic mutations in acute leukemia^8^, essential thrombocythemia^9^, and DS–AMKL^10^. Here, we report for the first time a monozygotic twin pair concordant for AMKL that besides a shared somatic *GATA1* mutation also shares a unique somatic mutation that must have arisen prenatally in an early preleukemic clone, and therefore is likely to be an early driver of the transformation into AMKL.

The monozygotic twins were monochorionic, diamniotic, and born by acute cesarean at 33.5 weeks of gestation due to an abnormal umbilical artery Doppler flow. Both neonates showed dysmorphic DS features, and the diagnosis was confirmed by conventional chromosome analysis showing a male karyotype with trisomy 21 in all analyzed cells. The twins did not present with TAM clinically, but at the age of 11 months, they simultaneously developed AMKL with 35–45% megakaryoblasts in the bone marrow. Both twins were found to have the same somatic *GATA1* duplication in exon 2, resulting in an N-terminally truncated protein consistent with the DS–AMKL diagnosis. Chromosome analysis and fluorescent in situ hybridization of cells obtained from bone marrow samples revealed a unique somatic translocation between chromosome 3 and 7: t(3;7)(q27;q32) in both twins (Fig. 1a, b, Supplementary Table S1). Cells with tetrasomy 21 were also found in both twins, whereas the other acquired chromosomal abnormalities were not shared (Supplementary Table S1). The twins were treated according to the international ML-DS protocol 2006^11^, had a parallel treatment response, gained full remission, and are both without disease 3 years after diagnosis.

**Fig. 1:**
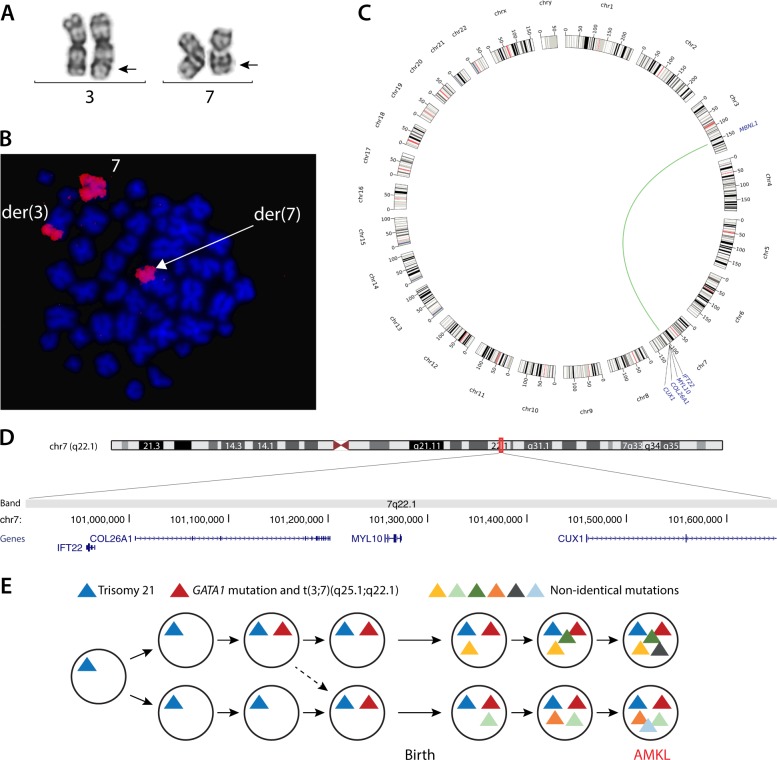
A shared somatic translocation in monozygotic twins with Down syndrome and acute megakaryoblastic leukemia (AMKL). **a** Partial karyogram of the somatic translocation showing the normal and the derivative chromosomes 3 and 7 (der(3) and der(7)) with the arrows indicating the approximate breakpoint positions. **b** The translocation was confirmed by fluorescent in situ hybridization with whole-chromosome paint using a red chromosome 7 probe, and by **c** mate-pair sequencing as illustrated with the green line in the circos plot. **d** The chromosome 7 breakpoint was found to delete/truncate four protein-coding genes as shown in the GRCh37 UCSC Genome Browser view of the deleted region associated with the 7q22.1 breakpoint. **e** The monozygotic twins are derived from a single fertilized egg with trisomy 21. The *GATA1* mutation and the somatic t(3;7)(q25.1;q22.1) translocation must have occurred in one of the twins, and a fraction of this clone was transferred to the other twin in utero. Subsequently several non-shared mutations arose in each twin, leading to synchronous AMKL development in both twins.

We mapped the shared somatic translocation by mate-pair sequencing (after written consent from the parents and as described in Supplementary Methods) with the purpose of identifying genes involved in the chromosomal rearrangement. As seen in Fig. 1c, we confirmed the two-way translocation and found no other structural rearrangements. The chromosome 3 breakpoint narrowed to a genomic region of 10.7 kb (chr3:152053805_152064541, hg19), which truncated intron 2 of the *MBNL1* gene (transcript variant 1). The chromosome 7 breakpoint involved a 755-kb deletion (chr7:100895760_101650853, hg19) encompassing four protein-coding genes: *IFT22*, *COL26A1*, *MYL10*, and the two first exons of *CUX1* (Fig. 1d). Thus, the karyotype was revised to seq[GRCh37] t(3;7)(q25.1;q22.1) g.[chr3:pter_cen_152053805::chr7:101650853_qter] g.[chr7:pter_cen_100895760::chr3:152064541_qter].

To search for additional mutations potentially involved in the leukemogenesis of these twins, we performed chromosomal microarray, whole-exome sequencing and RNA sequencing on DNA and RNA extracted from the bone marrow at the time of diagnosis, and chromosomal microarray and whole-genome sequencing of germline DNA extracted from peripheral blood after remission (Supplementary Methods). These genome-wide analyses were carried out as part of the STAGING research project approved by the regional ethical committee (H-15016782) and the Danish data protection agency (RH-2016-219, I-Suite no: 04804). No shared pathogenic variants were found besides the t(3;7)(q25.1;q22.1) translocation and the tetrasomy 21 mosaicism (Supplementary Fig. S1 and Tables S1–S3), but a constitutional *BRCA2* variant of unknown significance was found in both twins (the family pedigree was without breast and ovarian cancer in first to third-degree relatives). However, we found a number of non-shared somatic variants in each twin, e.g., duplication 1q and a *JAK3* missense variant in Twin A, and mosaicism for trisomy 8 and a frameshift variant in *RAD21* in Twin B (Supplementary Table S1). We screened for fusion transcripts on RNA-sequencing data and found none generated by the t(3;7)(q25.1;q22.1) translocation (see Supplementary Information). Gene expression of leukemic cells revealed a slightly higher expression of *JAK3* in Twin A compared with Twin B, whereas no noticeable differences were found for, e.g., *RAD21*, *CUX1*, and *MBNL1* (Supplementary Table S4).

Several observations support that the acquired t(3;7)(q25.1;q22.1) translocation is an important leukemogenic step; (1) since both the unique translocation and the specific *GATA1* mutation are shared, they both must have arisen in one twin and thereafter been transferred to the other twin in utero; (2) no other pathogenic variants were shared in these twins who developed AMKL at the same time, had parallel disease progression, and the same treatment response; (3) chromosome 7 and 7q abnormalities are recurrent somatic aberrations, e.g., found in ~6% of patients with DS–AMKL^4^. We therefore mapped the chromosomal breakpoints to search for 7q genes driving this synchronous AMKL progression in the twins. Indeed, one of the four truncated/deleted genes on chromosome 7q was the transcription factor, *CUX1*: a tumor-suppressor gene in which inactivating monoallelic mutations have been found to promote tumorigenesis in myeloid malignancies^12,13^. The other three deleted/truncated genes on chromosome 7 are without a known role in leukemia. The truncated gene on chromosome 3, *MBNL1*, is a splicing regulator, and has been found in one study to be deleted in pediatric acute myeloid leukemia, often in combination with a *ZEB2* deletion^14^, and the loss-of-function mutation of *MBNL1* could therefore also be involved in DS–AMKL development. An extra supernumerary chromosome 21 was found in all cells containing the translocation in both twins, indicating that gain of chromosome 21 may be an early contributing factor too. Trisomy 8, 1q duplication, and pathogenic variants in *JAK3* and *RAD21* have previously been reported in DS–AMKL^4–6,15^, but since they were only found in one twin each, these variants must have occurred later in the leukemic development than the t(3;7)(q25.1;q22.1) translocation (as illustrated in Fig. 1e).

This is the first report of a somatic translocation involving *CUX1* in patients with DS–AMKL. Based on the shared occurrence in the twins combined with studies showing chromosome 7q involvement in DS–AMKL^4^ and the role of *CUX1* in myeloid malignancies^12,13^, we propose that an acquired loss-of-function mutation in *CUX1* can be a critical early, and even prenatal, somatic event that drives a *GATA1*-mutated clone into AMKL in patients with Down syndrome.

## Supplementary information

Supplementary Information

Fig S1

Table S1

Table S2

Table S3

Table S4
