# Supplementary material for: A shared somatic translocation involving CUX1 in monozygotic twins as an early driver of AMKL in Down syndrome
Source: Blood Cancer J. 2020 Mar 3;10(3):27. doi: 10.1038/s41408-020-0293-6 (PMC7054393; doi:10.1038/s41408-020-0293-6)
Supplement: Supplementary file 1 — Supplementary Information [file 41408_2020_293_MOESM1_ESM.docx]

**Supplementary Information**

A shared somatic translocation involving *CUX1* in monozygotic twins as an early driver of AMKL in Down syndrome

By Iben Bache, Karin Wadt, Mana M. Mehrjouy, Maria Rossing, Olga Østrup, Anna Byrjalsen, Niels Tommerup, Marlen Metzner, Paresh Vyas, Kjeld Schmiegelow, Birgitte Lausen, Mette K Andersen

**This supplementary information contains** Supplementary Methods (page 2-4), Supplementary Appendix S1: Panel of 314 cancer-predisposing/candidate cancer genes (page 5), Supplementary References (page 6), and Supplemental Figure and Table Legends (page 6).

**Supplementary Methods**

Chromosome analysis and fluorescent in situ hybridization (FISH): Metaphase chromosomes from cultured peripheral blood lymphocytes and bone marrow cells were prepared according to standard protocols, G-banded and karyotyped. Metaphase FISH was performed using whole chromosome painting probes for chromosomes 3 and 7 (Vysis, Abbott, Illinois, US). All cytogenomic results are written in accordance with An International System for Human Cytogenomic Nomenclature ISCN 2016^1^.

DNA and RNA: DNA was purified from peripheral blood and from bone marrow using a QIAamp DNA Mini Kit (Qiagen, Hilden, Germany) according to the manufacturer's instructions. RNA was extracted from bone marrow using standard TRIZOL protocol with subsequent QC steps.

Mate-pair sequencing: Mate-pair libraries were prepared using the Mate Pair Library v2 kit (Illumina, San Diego, CA, US), and paired-end sequenced (2x150 bases) on a Genome Analyzer IIx (Illumina). Paired reads were aligned to the GRCh37/hg19 reference genome, and reads that aligned to different chromosomes or with unexpected strand orientations were extracted and analyzed to identify chromosomal rearrangements as previously decribed^2^.

Chromosomal microarray: DNA from both bone marrow and peripheral blood was analyzed with SNP array using CytoScan assay (ThermoFisher, Waltham, MA, US). Assay was performed according to the manufacturer´s instructions. The .CEL files from the CytoScan assay were imported into NEXUS v10.0 (BioDiscovery, El Segundo, CA, US) and used for the analysis and visualization of copy number variations and alterations (CNVs, CNAs) using NCBI Build 37 as reference. Briefly, the sample were pre-processed by systematic correction (Quadratic), probes were re-centered by median and applying mean of Combine Replicates Between Arrays. Subsequently, data were processed by SNP-FASST2 Segmentation with significance threshold of 1.0E-8 and max contiguous probe spacing of 1000 Kbp with minimum of 3 probes per segment.

Whole-exome sequencing: SureSelect All Exon Kit v5 (Agilent Technologies, Santa Clara, CA, USA) was used for exome enrichment. Sequencing was conducted using the HiSeq2500 platforms (Illumina). In brief, 1 μg of genomic DNA was fragmented on a Covaris S2 (Woburn, MA, USA) to an average size of 250 bp. Trimming, 3’-adenylation and adaptor ligations were done on a Sciclone G3 robot (Perkin Elmer, Waltham, MA, USA) using Illumina-compatible KAPA library DNA adaptors (Roche Diagnostics, Basel, Switzerland). Sequencing was performed as paired-end sequencing, 2 × 101 bases or 2 × 151 bp, resulting in approximately 100 mol/l paired-end reads. Sequencing data were processed using Consensus Assessment of Sequence and Variation (CASAVA) v1.8.2 (Illumina). Generated fastq files were uploaded to CLC bio (Genomics Workbench v2.5.1; Qiagen) for trimming of the last 3’ base, mapping and variant calling. All the algorithms applied to the samples were custom made by CLC bio. The reads were mapped to the GRCh37/hg19 reference genome with custom algorithm CLC4 (<https://www.qiagenbioinformatics.com/products/clc-genomics-workbench/>) for a comparison between the CLC4 algorithm and the most commonly known aligners. Quality control of the target sequencing was performed on the read mappings. The variant calling was performed by a Maximum Likelihood approach on a Bayesian model. Variants were called with a minimum of 9x coverage, 3 counts and 1% frequency. Variants were further filtered using the Ingenuity Variant Analysis tool (Qiagen), excluding the low-quality scores and variants (>1% in background population),

Whole-genome sequencing: was performed by an external collaborator (Norwegian Sequencing Center) and conducted on the HiSeqX Illumina platform using paired-end sequencing of 150 bp reads with 30X depth and mapped to the GRCh37/hg19 reference genome. Pipeline: GATK-based Sentieon bioinformatic pipeline. VarSeq was used for SNP and indel calling. All variants with a frequency below 1% in 314 cancer predisposition genes or candidate cancer genes (listed in Supplemental Appendix 1) were called. For *ATM* and *CHEK2,* variants below 5% were called.

RNA-sequencing: was performed using Illumina’s TruSeq Stranded Total RNA Library Prep Kit and paired-end sequencing was performed to gain an average output of 50–100M reads. We used standard clinical pipeline for expression-data and calculated transcripts per million (TPM) for each gene. TPM_i = 10^6 * (N_i / L_i) / [Σ_j (N_j / L_j)], where N_i is the number of reads coming from gene i and L_i the length of gene i, and then divide by the sum of this quantity for all genes and in the end multiplied by 1 million. FusionMap was used for the screening of fusion transcripts^3^.

Data sharing statement. For original data from mate-pair sequencing, chromosomal microarray, whole-exome sequencing, RNA-sequencing and whole-genome sequencing, please contact [ibache@sund.ku.dk](mailto:ibache@sund.ku.dk).

**Supplementary Appendix S1: Panel of 314 cancer-predisposing/candidate cancer genes**

*ABCB11, ABRAXAS1, ACD, ACTRT1, ADA, AIP, AKT1, ALK, ANKRD26, APC, ARID5B, ARMC5, ASXL1, ATG2B, ATM, ATR, AXIN2, A2ML1, BAP1, BARD1, BLM, BMPR1A, BRAF, BRCA1, BRCA2, BRF1, BRIP1, BUB1 , BUB1B, BUB3, CABLES1, CASP9, CBL, CDC73, CDH1, CDH23, CDK4, CDKN1A, CDKN1B, CDKN1C, CDKN2A, CDKN2B, CEBPA, CEP57, CHEK2, CIP2A, COL7A1, CREBBP, CTC1, CTNNA1, CTNNB1, CTR9, CYLD, DDB2, DDX41, DHCR7, DHX34, DICER1, DIS3L2, DKC1, DNAJC21, DNMT3A, DOCK8, EDC4, EFL1, EGFR, ELANE, EP300, EPCAM, ERBB2, ERCC1, ERCC2, ERCC3, ERCC4, ERCC5, ERCC6, ERCC8, ERCC6L2, ETV6, EXO1, EXT1, EXT2, EZH2, FAH, FAN1, FANCA, FANCB, FANCC, FANCD2, FANCE, FANCF, FANCG, FANCI, FANCL, FANCM, FAS, FBXW7, FGFR2, FGFR3, FH, FLCN, FMR1, FOCAD, GAB2, GATA1, GATA2, GATA3, GBA, GFI1, GJB2, GLI3, GNA11, GNAQ, GNAS, GPC3, GPR161, GREM1, GPR101, GSKIP, G6PC3, H19, HABP2, HAX1, HFE, HMBS, HNF1A, HOXB13, HRAS, IKZF1, INHA, INHBA, IPMK, ITK, KCNQ1OT1, KDM1A, KDM3B, KIF1B, KIT, KLLN, KRAS, L2HGDH, LIG4, LZTR1, MAD2L2, MAP2K1, MAP2K2, MAX, MBD4, MC1R, MDH2, MECOM, MEN1, MET, MITF, MLH1, MLH3, MRE11, MSH2, MSH3, MSH6, MTAP, MUTYH, NAF1, NBN, NDRG4, NF1, NF2, NFATC4, NFIX, NHP2, NOP10, NPM1, NRAS, NSD1, NTHL1, NTRK1, NYNRIN, PALB2, PARN, PAX5, PDCD4, PDGFRA, PDGFRB, PHOX2B, PIK3CA, PIP4K2A, PMS1, PMS2, POLD1, POLE, POLH, POT1, POU6F2, PPM1D, PRF1, PRKAR1A, PRSS1, PTCH1, PTCH2, PTEN, PTPN11, PTPN12, RAD50, RAD51, RAD51B, RAD51C, RAD51D, RAF1, RASA2, RB1, RBBP6, RECQL, RECQL4, REST, RET, RFWD3, RHBDF2, RINT1, RIT1, RMRP, RNF43, RPL3, RPL5, RPL10, RPL10A, RPL11, RPL15, RPL19, RPL23, RPL26, RPL27, RPL31, RPL34, RPL35A, RPL36, RPLP0, RPS7, RPS10, RPS11, RPS15, RPS17, RPS19, RPS20, RPS24, RPS26, RPS27, RPS27A, RPS28, RPS29, RRAS, RTEL1, RUNX1, SAMD9, SAMD9L, SBDS, SDHA, SDHAF2, SDHB, SDHC, SDHD, SEC23B, SERPINA1, SETBP1, SH2B3, SH2D1A, SHOC2, SLC25A11, SLX4, SMAD4, SMAD9, SMARCA2, SMARCA4, SMARCB1, SMARCE1, SMO, SOS1, SOS2, SPINK1, SPRED1, SRP72, SRY, STAT3, STK11, STN1, SUFU, TERC, TERF2IP, TERT, TFAP2A, TGFBR1, TINF2, TMEM127, TNFRSF13B, TP53, TP53AIP1, TRIM28, TRIM37, TRIP13, TSC1, TSC2, TSR2, UBE2T, UROD, USB1, VHL, VPS13C, WAS, WRAP53, WRN, WT1, XPA, XPC, XRCC2, XRCC3*

**Supplementary References**

1 McGowan-Jordan J, Annet S, Schmid M. *ISCN: An International System for Human Cytogenomic Nomenclature (2016)*. Karger: Basel, Switzerland, 2016.

2 Halgren C *et al.* Haploinsufficiency of CELF4 at 18q12.2 is associated with developmental and behavioral disorders, seizures, eye manifestations, and obesity. *Eur J Hum Genet* 2012; **20**: 1315–1319.

3 Ge H *et al.* FusionMap: Detecting fusion genes from next-generation sequencing data at base-pair resolution. *Bioinformatics* 2011; **27**: 1922–1928.

**Supplementary Figure and Table Legends:**

**Fig. S1 -** Somatic copy number variants detected by chromosomal microarray.

**Table S1** - Genetic findings in a pair of monozygotic twins with Down syndrome and Acute Megakaryoblastic Leukemia.

**Table S2** - Somatic variants detected by whole-exome sequencing.

**Table S3** - Germline variants detected by whole-genome sequencing of DNA.

**Table S4** - Expression of selected genes in leukemic cells.
