# Supplementary figures and images for: A shared somatic translocation involving CUX1 in monozygotic twins as an early driver of AMKL in Down syndrome
Source: Blood Cancer J. 2020 Mar 3;10(3):27. doi: 10.1038/s41408-020-0293-6 (PMC7054393; doi:10.1038/s41408-020-0293-6)

**Fig. S1**

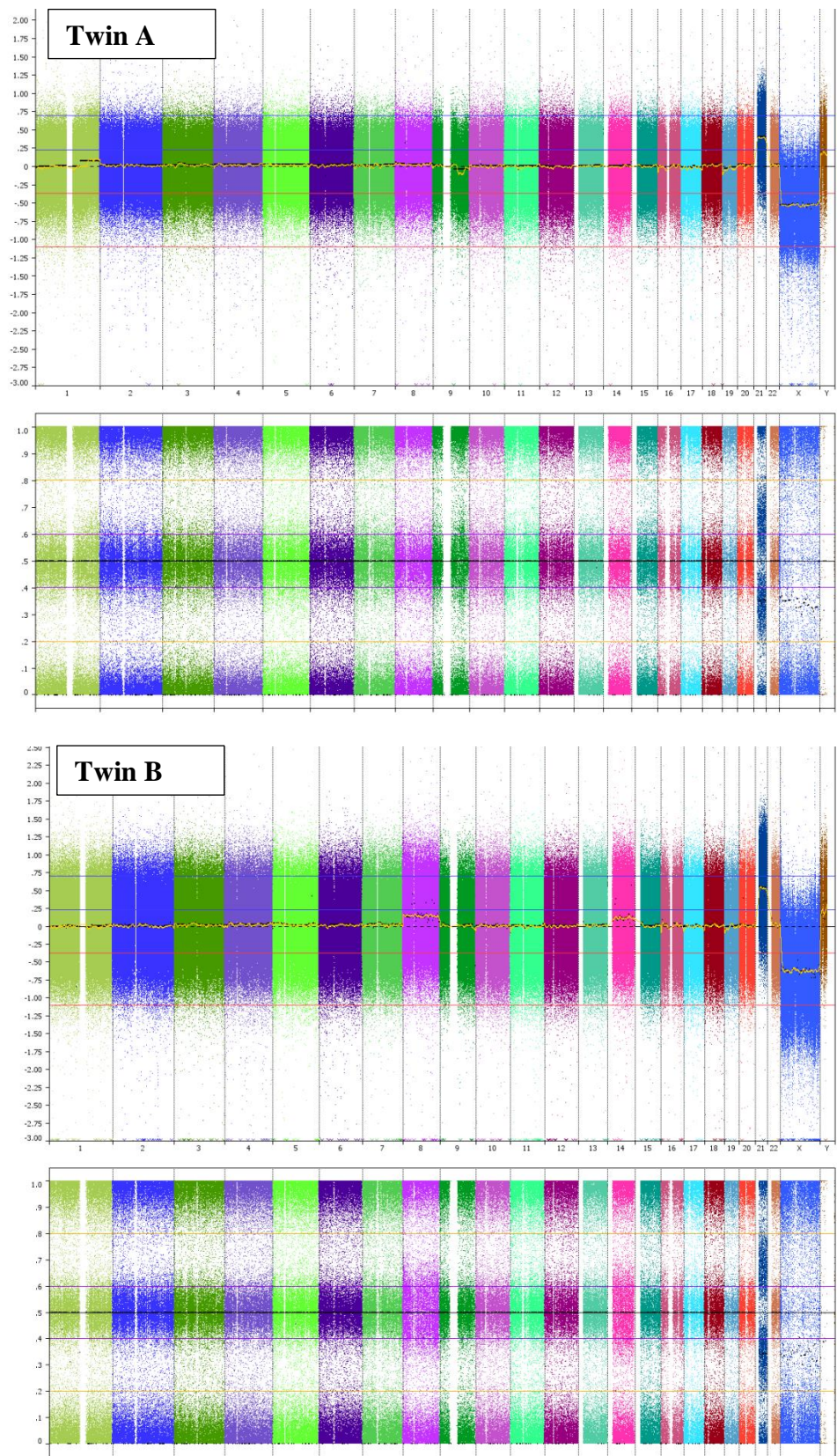

Supplement: Supplementary file 2 — Fig S1 [file 41408_2020_293_MOESM2_ESM.pdf]
